# In-house three dimensional-printed cutting guides improve surgical accuracy in children who underwent resection of malignant bone tumours of lower limb and reconstruction with allograft

**DOI:** 10.1007/s00264-026-06773-8

**Published:** 2026-03-17

**Authors:** Eiji Nakata, Giulia Alessandri, Grazia Chiara Menozzi, Ayano Aso, Toshifumi Ozaki, Giovanni Trisolino, Davide Maria Donati, Costantino Errani

**Affiliations:** 1 Department of Orthopedic Surgery, Okayama University Hospital, Okayama city, Japan; 2 Orthopaedic Service, Musculoskeletal Oncology Department., IRCCS Istituto Ortopedico Rizzoli, Bologna, Italy; 3 Unit of Pediatric Orthopedics and Traumatology, IRCCS Istituto Ortopedico Rizzoli, Bologna, Italy

**Keywords:** Massive bone allograft, In house 3-dimensional-printed patient-specific instruments, Malignant bone tumours, Lower limb, Children

## Abstract

**Aims:**

This study evaluated the accuracy of resection of bone tumours and the fit between host bone and massive bone allograft (MBA) in children with malignant bone tumours of lower limb who underwent surgery using in-house 3-dimensional (3D)-printed patient-specific instruments (PSIs) for tumour resection and graft-specific instruments (GSIs) for shaping the MBA.

**Methods:**

This retrospective study included seven children (3 males, 4 females; median age 13) with malignant bone tumours of the lower limb who underwent intercalary resection and reconstruction with MBA between September 2023 and March 2025 using in-house designed 3D-printed PSIs and GSIs. Tumours were located in the femur (5 children) and tibia (2 children). We analysed the accuracy of bone resection, complications of reconstruction, and function of patients.

**Results:**

All resections achieved R0 margins. The median planned resection length was 16.5 cm versus 16.8 cm actually resected (median difference 0.2 cm). Bone union was achieved in 13 of 14 (92.9%) osteotomy sites. Bone union was faster at metaphyseal junctions (median 5.9 months) than diaphyseal junctions (median 8.4 months) (*p* = 0.01). One of the osteotomy sites (7.1%) had a delayed union requiring secondary bone grafting. The median Musculoskeletal Tumour Society score was 30 at the last follow-up.

**Conclusion:**

3D-printed PSIs and GSIs appear to enhance the accuracy of bone tumour resection and host bone-MBA fit, thereby reducing the risks of inadequate margins and non-union, respectively.

## Introduction

Recently, 3-dimensional (3D)-printing technology was clinically applied including the creation of anatomical models, patient-specific cutting guides, and patient-specific prostheses/instruments in the orthopaedic oncological surgery [1–3]. Several retrospective studies showed that 3D-printed PSIs enabled more precise multiplanar osteotomies in complex anatomical locations, such as the pelvis and juxta-articular region, reducing operative time and blood loss [4–7]. Moreover, the use of graft-specific instruments (GSIs) can shape the MBA to adequately match the bone defect following tumour resection [8–10]. Achieving near-perfect congruency between host bone and massive bone allograft (MBA) may increase the contact surface area, which may facilitate bone union [11]. Previous monocentric retrospective studies focusing on the accuracy of bone resection in patients with pelvic and extremity bone tumours using 3D-printed PSIs reported that the difference between the length of preoperatively planned bone resection and the resected specimens ranged from 2.3 mm to 8.4 mm [12]. However, these reports have largely focused on resection accuracy, often in anatomically complex pelvic or juxta-articular locations, and have rarely addressed intercalary reconstructions with MBA, particularly in children [8, 10]. To date no studies reported the accuracy of bone resection and clinical outcome focusing on intercalary resection and reconstruction with MBA using in-house 3D-printed PSIs both for the resection and the reconstruction with MBA in children with malignant bone tumours of lower limb. Therefore, we conducted a retrospective study to evaluate the accuracy of bone resection and reconstruction with MBA following intercalary resection using in-house 3D-printed PSIs in children with malignant lower limb bone tumours, as well as to assess the associated union rates, complications, and functional outcomes.

## Materials and methods

### Patient characteristics

A retrospective study was conducted on children with malignant bone tumours of the lower limb who underwent intercalary resection of bone tumours and reconstruction with MBA. All children were treated at our institution between September 2023 and March 2025. Inclusion criteria were children with malignant bone tumours of the femur or tibia who underwent intercalary resection and reconstruction with MBA using in-house 3D-printed PSIs and GSIs. Exclusion criteria were adult patients or children who underwent hemi-cortical resection or radiotherapy at the surgical site. During the study period, seven children with malignant bone tumours of the lower limb underwent intercalary resection of the tumour and reconstruction with MBA (Table 1). The patient population consisted of three males and four females, with a median age of 13 years (range 9–17 years). The tumours were located in the femur in five children and in the tibia in two children. Pathological analysis of resected specimens revealed Ewing sarcoma in five children and osteoblastic osteosarcoma in two children. By the American Joint Committee on Cancer staging system, all children were classified as stage IIA. All children received chemotherapy, consisting of a combination of neoadjuvant and adjuvant chemotherapy. Median follow up period was 10.0 months (range 6.2–21.9 months). Surgical margins were evaluated according to the American Joint Committee on Cancer (AJCC) residual tumour classification and categorized as R0 (no residual tumour), R1 (microscopic residual tumour cells at the inked margin), or R2 (macroscopic residual tumour cells) [13].

**Table 1:** Patient characteristics

Case	Sex	Age at presentation	Location	Diagnosis	Tumor Size (cm)	Length of Planned resection (cm)	Length of resected bone (cm)	Complication	MSTS
1	M	9	Proximal Femur	Ewing sarcoma	3.9	15.6	16	Delayed healing	30
2	M	11	Proximal Femur	Ewing sarcoma	2.9	16.5	16.7	-	30
3	F	17	Distal Femur	Ewing sarcoma	5.2	14.7	15	-	26
4	F	16	Proximal Tibia	Osteosarcoma, osteoblastic	5.1	15	15	-	30
5	M	13	Distal Tibia	Ewing sarcoma	3.1	22	22	-	30
6	F	9	Distal Femur	Osteosarcoma, osteoblastic	4.4	18.5	18.7	-	23
7	F	17	Proximal Femur	Ewing sarcoma	5.2	23.5	23.5	-	29

*M* male, *F* female

*MSTS* musculoskeletal tumour society

### Procedure of in-house 3D-printed patient-specific cutting guides

All procedures were planned, simulated, and executed through a close collaboration between the Paediatric Orthopaedics Unit and the Orthopaedic Oncology Unit, within a study protocol approved by the Ethics Committee (NCT05700526). The protocol, which was initially authorized for the management of severe musculoskeletal deformities in paediatric patients, was subsequently amended to include massive resections in paediatric oncologic cases as well. The in-house 3D-printing workflow is well established and thoroughly validated [14, 15].

### Preoperative planning

**Fig. 1:**
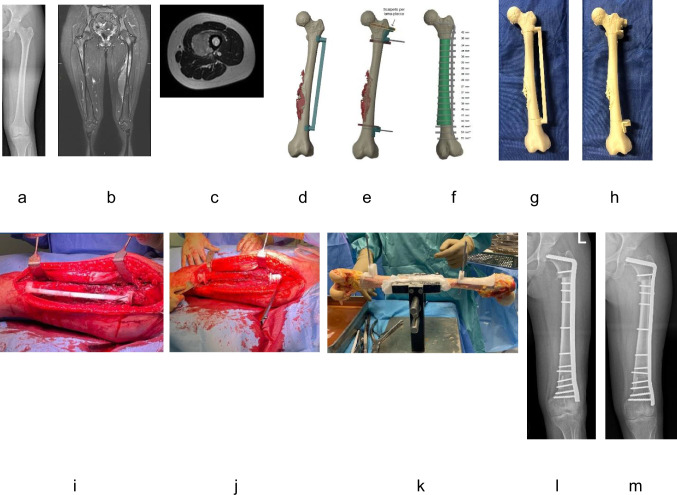
An example of preoperative planning, design and 3D printing, and intraoperative use of patient-specific cutting guides is shown. The patient was a 17-year-old female with Ewing sarcoma in the left femur. Plain radiographs demonstrated a lytic lesion in the femur (Fig. 1a). Magnetic Resonance Imaging was utilized to evaluate the extraosseous size and intramedullary extension of the tumour (Fig. 1b, c). The models were validated by the surgical team and used for virtual surgical planning in Blender, where resection planes ensuring safe margins (> 1 cm) were defined by an engineer in collaboration with the surgeon (Fig.  1 d, e). Donor CT scans of MBA were segmented to generate 3D model, which was aligned with the patient’s anatomy in Blender to optimise host-graft contact (Fig. 1f). Anatomical models reproduced the affected bone and the selected donor segment at real scale (Fig. 1 g, h) to support surgical planning and verify the proper fitting of the guides. Intraoperatively, adequate bone was exposed to allow secure placement of the patient-specific cutting guide, which was fixed to the native bone with Kirschner wires and osteotomies were then performed (Fig. 1i, j). The graft-specific cutting guide was subsequently applied to the donor bone to harvest the intercalary graft (Fig. 1 k), which was then positioned at the resection site and stabilized with internal fixation (Fig. 1 l). The time to bone union in the metaphyseal and diaphyseal junction was 3.4 months and 6.2 months, respectively (Fig. 1 m)

Preoperative imaging included X-ray, Computed Tomography (CT) scans and Magnetic Resonance Imaging (MRI) to assess bone anatomy and tumour margins (Fig. 1a, b, c) [16]. MRI was utilized to evaluate the extraosseous size and intramedullary extension of the tumour (Fig. 1b, c). CT scans and MRI datasets were segmented to obtain 3D models of bone and tumour tissues and registered to align both structures using Materialise Mimics (Materialise, Leuven, Belgium). The models were validated by the surgical team and used for virtual surgical planning into the open-source software Blender (Blender Foundation), where resection planes ensuring safe margins (> 1 cm) were defined by an engineer in collaboration with the surgeon (Fig. 1d, e). The team refined the surgical approach, determined safe cutting levels and optimal osteotomy trajectories, and planned reconstruction strategy using plates. CT scans of multiple candidate donor grafts were segmented following the same workflow used for the patient bone to generate 3D models. The donor models were then aligned to the recipient anatomy in Blender and the graft providing the best anatomical match was selected to optimise host–graft contact (Fig. 1f). The resection cutting planes defined on the patient model were transferred to the donor bone, serving as geometric references for GSI design. Several iterative planning sessions between the surgeon and engineer allowed the simulation of resection scenarios, including evaluation of the surgical approach and incision placement, leading to the selection of the most feasible and accurate solution.

### Design and 3D printing

The design and 3D-printing of patient-specific models and instruments were categorized into three groups: anatomical models of the patient and donor, patient-specific cutting guides, and graft-specific cutting guides. Anatomical models reproduced the affected bone and the selected donor segment at real scale (Fig. 1g, h) to support surgical planning and verify the proper fitting of the guides. Cutting guides were developed to improve precise osteotomy and included fixation holes positioned to avoid tumour contact. In cases requiring blade plates, additional slots allowed chisel insertion for plate osteosynthesis. Both patient- and donor-specific cutting guides featured support bases to stabilize the saw blade during osteotomy. Anatomical models were designed in Blender and fabricated using FDM 3D printing technology (Bambu Lab X1C, Bambu Lab, Shenzhen, China) with polylactic acid (PLA) filament (FiloAlfa®). Cutting guides were designed in PTC Creo Parametric (PTC Inc., Boston, MA, USA) based on the digital bone surface and on the predefined osteotomy planes, using standard Computer-Aided Design operations and printed with FDM technology (Qidi i-Mates, Qidi Tech, Zhejiang, China) using PLA Crystal filament (Fillamentum®). The main 3D-printing parameters are summarized in Table 2. Segmentation of a lower limb from CT and MRI datasets required approximately  six to eight h in total, considering both patient and donor imaging set. Preoperative planning with the surgeon typically involved three meetings, each lasting one to 1.5 h, during which medical images, digital 3D models, anatomical models, and prototypes of cutting guides were reviewed. The design of patient-specific cutting guides required approximately 1.5–two h per guide, with each developed sequentially using geometric reference features from the previous one to ensure consistency. For 3D printing, anatomical bone models required on average  three to four h per model (6–8 h per case), while definitive cutting guide, printed in sterilizable material, required on average six h. Post-processing included one h of annealing, as recommended in the material datasheet, and an additional one h for support removal and preparation for sterilization. The design and fabrication of patient-specific instruments, including both cutting guides and anatomical models, is a time-consuming process, requiring on average approximately 27.5 to 33.5 non-continuous hours. All cutting guides were sterilized up to  two days before surgery and delivered to the operating room on the day of the intervention.

**Table 2:** Main 3D-printing parameters

Parameter	FiloAlfa® PLA	Fillamentum® PLA Crystal
Printing Temperature	200 °C	220 °C
Heated Bed Temperature	60 °C	60 °C
Nozzle Diameter	0.4 mm	0.4 mm
Layer Thickness	0.2 mm	0.2 mm
Printing Speed	60 mm/s	60 mm/s
Travel Speed	200 mm/s	150 mm/s
Infill Density	15% Lightning	100% Line
Flow	100%	100%
Cooling	Yes	Yes
Support	Yes	Yes

### Intraoperative use of patient-specific cutting guides

Adequate bone was exposed to allow secure placement of the PSI, which was fixed to the native bone with Kirschner wires (Fig. 1i, j). Positioning was verified by fluoroscopy and direct inspection. In patients requiring a reconstruction using blade plate, a chisel groove was created thorough additional guide slots. Osteotomies were then performed using power oscillating saws, and residual soft tissues were carefully dissected to allow en bloc removal of both bone and guide. The GSI was subsequently applied to the donor bone to harvest the intercalary graft (Fig. 1k), which was then positioned at the resection site and stabilized with internal fixation (Fig. 1l, m).

### Clinical and radiographic follow-up

Children underwent radiographic follow-up every three months until bone union of MBA was achieved. Bone union was defined based on modified Radiographic Union Score for Tibia (mRUST) score of 9 on both anteroposterior and lateral radiographs [17]. Bone union was independently assessed by EN and AA who were not involved in the index surgical procedure. In cases of discrepancy between the two assessments, a consensus reading was performed through joint review to reach a final decision. The time of bone union was also assessed.

### Assessment of study outcomes

The length of the resected specimen was compared with the length of preoperatively planned bone resection to investigate the accuracy of patient-specific cutting guides [8]. The resected specimen was radiographed, and its length was measured based on the X-ray image. In case where X-rays was not taken, the value recorded in the pathological report was used as the resection length. The type and frequency of surgical complications were documented. The functional results were evaluated according to the Musculoskeletal Tumour Society (MSTS) 93 score [18]. The Mann–Whitney U test was used to analyse continuous parameters. For all analyses, associations were considered significant at a *P* < 0.05, and the Bell Curve for Excel (Social Survey Research Information Co., Ltd., Tokyo, Japan) was used.

### Ethical approval

Ethical approval for this study was obtained from the Ethic Committee of Area Vasta Emilia Centro (CE AVEC: 301/2022/Sper/IOR).

## Results

### 3D-printed cutting guides

Cutting guides were used in all patients included in this study. On average, three to four cutting guides were employed per case, accounting for both the patient and the donor graft. No mechanical issues, fractures, deformations, or other alterations affecting guide usability were reported.

### Surgical treatment, margins, and accuracy

Intercalary resections were performed using PSIs and the reconstruction was made with an intercalary MBA shaped using GSI in all children. In two children, 3D-printed PSIs were designed with an integrated blade groove to guide also the osteosynthesis with a blade plate. Internal fixation was achieved with a plate in all children. The median surgical time was 232 min (range 195–264 min). The surgical margins were R0 in all children. The median length of preoperatively planned bone resection and resected specimen was 16.5 cm (range 15.0–23.5 cm) and 16.8 cm (range 16.0–23.5 cm), respectively. The median difference in bone length was 0.2 cm, ranging from 0–0.4 cm (*p* = 0.81) (Table 1). No local recurrence or distant metastases were observed at the latest follow-up.

### Bone union

Bone union was achieved in 13 out of 14 (92.9%) osteotomy sites, with only one non-union at the diaphyseal junction. The median time to bone union in the metaphyseal and diaphyseal junction was 5.3 months (range 3.0–6.5 months) and 8.7 months (range 6.2–10.8 months), respectively (*p* = 0.01).

### Complications

One of seven children (14.3%) presented delayed union at the diaphyseal junction which was successfully treated with a strut allograft six months after the initial surgery. No other complications were observed.

### Functional outcomes

At the latest follow up, the median MSTS Score was 30 (range, 23–30).

## Discussion

Previous studies showed that 3D-printed patient-specific cutting guides enabled accurate multiplanar osteotomies in complex areas like the pelvis while reducing operative time and blood loss [1]. In addition, these 3D-printed patient-specific cutting guides facilitated shaping MBA to match tumour defects, potentially enhancing bone union [10]. In the present series, the use of in-house 3D-printed patient-specific cutting guides designed for both tumour resection and graft reconstruction enabled accurate resection with negative margins and facilitated the accuracy of fit between the host bone and MBA following intercalary resection in children with malignant bone tumours of the lower limb.

### Limitations

This study has some limitations. First, the sample size was limited due to the novelty approach of the application of 3D printing technology in paediatric orthopaedic oncology. Second, the follow-up period for children in this study was relatively short, precluding full assessment of long-term oncological outcomes and late complications. However, evaluation of surgical margins, correct planned lengths of resection of the tumour and reconstruction with MBA and the union of the osteotomy site was feasible, as these parameters do not require long follow-up such as for oncological complication. Third, there is a lack of a control group in this study. However, because sarcomas in children are uncommon, it is very difficult to conduct a prospective study comparing the accuracy of 3D-printed cutting guide and free hand technique in children with malignant bone tumours of lower limb who receive joint‐preserving surgery by intercalary resection and reconstruction with MBA. Therefore, the present findings can only be interpreted in comparison with previously published studies analysing intercalary reconstruction with MBA in children with bone tumours who underwent free hand intercalary resection. Fourth, length of resected specimen was primarily determined using postoperative radiographs, and pathological reports were consulted when radiographs were unavailable. Radiographic measurements are susceptible to magnification errors, whereas pathological measurements may be influenced by residual soft tissue and formalin-induced shrinkage. This heterogeneity in measurement methods may have introduced bias in the assessment of resection accuracy. Although the discrepancy between the planned and actual resection lengths was small, these methodological differences should be taken into account when interpreting the precision of the cutting guides. Finally, the authors attempted to clarify the context of their findings by comparing the results with historical control groups reported in the literature. However, inter-study comparisons may be influenced by differences in patient selection, surgical techniques, and outcome assessments; therefore, any definitive conclusions regarding superiority should be drawn with appropriate caution.

### Clinical challenges 

Malignant bone tumours of the lower limb in children requiring intercalary resection and reconstruction with MBA represent a technically demanding surgery [14]. The main challenges include achieving oncologically safe margins while preserving joint function, obtaining precise multiplanar osteotomies in growing bone, and ensuring optimal host–graft congruency to reduce the risks of non-union, fracture, and implant failure [1]. Conventional freehand techniques or intraoperative navigation systems may still result in geometric inaccuracy and prolonged operative time [19, 20]. The present in-house 3D-printing workflow enables meticulous preoperative planning by repetitive surgeon–engineer collaboration, accurate transfer of resection planes to both host bone and allograft, and preoperative verification of guide fitting. This structured approach improves resection precision, enhances graft fit, and may contribute to lower complication rates and improved functional outcomes in this complex paediatric setting [1].

### Surgical treatment, margins, and accuracy

Previous monocentric retrospective studies showed that 3D-printed patient-specific cutting guides achieved negative margin of resection of tumours in long bones in most of the patients (90.1–100%) (Table 3) [5–7]. Previous monocentric retrospective studies focusing on the accuracy of bone resection reported that the difference between the length of preoperatively planned bone resection and the resected specimens ranged from 2.3 mm to 8.4 mm in patients with pelvis and extremity bone tumours using 3D-printed patient-specific cutting guides [8, 12, 21]. In a monocentric retrospective study, 29 patients with malignant primary bone tumour of the pelvis and extremity underwent tumour resection using a custom-made 3D-printed patient-specific cutting guide: the accuracy of bone resection was evaluated by comparing the planned length and the length from the osteotomy site to those of the tumour in the actual resected bone specimens, revealing a difference of 1–3 mm [22]. In our study, all resections achieved R0 margins and the median deviation between planned and actual resection length was only 0.2 cm. These findings are consistent with previous studies. The limited discrepancy observed in our cohort suggests that the integration of the in-house 3D-printing workflow contributes to enhanced geometric accuracy.

**Table 3:** Summary of clinical studies of 3D-printed patient-specific cutting guides in orthopaedic oncology

Authors	Patient Number	Site	Histology	Usage of 3D Printing Techonology	Negative Margin	Local Recurrence	Complication	MSTS (0–30)	Accuracy of Osteotomy
Wang et al. [4]	33	Femur Tibia	CS, GCT, OS	G	90.9%	9.1%	12.1%	Median: 28	NA
Ma et al. [7]	8	Femur	OS	M/G/R	100%	0%	NA	Average: 27	NA
Wong et al. [5]	3	Femur Tibia	EWS, OS	G/I	100%	0%	0%	Median: 29	The mean maximum deviation errors of the nine achieved bone resections: 1.64 ± 0.35 mm
Park et al. [8]	12	Pelvis, Sacrum, Extremity	CS, Meta, OS, STS	G, G/R, or G/I	100%	8.3%	NA	NA	The mean cutting error for the shortest margin: 1.2 (range 0 to 3) mm. The mean cutting error for the greatest margin: 1.4 (range 0 to 3) mm. The maximal cutting error: 3 mm
Müller et al. [10]	12	Pelvis, Extremity	CS, EWS, OS	G/R	91.7%	0%	NA	NA	The error of osteotomy: 0.74 ± 0.96 mm-3.60 ± 2.46 mm
Dong et al. [6]	17	Pelvis, Femur, Tibia	EWS, Meta, OS, CS, GCT	M/G, M/G/R, M/G/I, M/G/R/I	93.8%	0%	47.1%	Average: 24	Average difference between planned resection length and resection length: 8.35 mm
Gasparro et al. [9]	6	Femur Tibia	CS, EWS, OS	G/R	100%	0%	50%	NA	NA

*M* model, *G* resection guide, *R* reconstruction guide, *I* implant

*CS* chondrosarcoma, *EWS* ewing sarcoma, *GCT* giant cell tumor, *Meta* metastatic tumors, *OS* osteosarcoma, *NS* not significant, *STS* soft-tissue sarcomas, *NA* not available

### Bone union

Intercalary reconstruction with MBA results in non-union in 6–43% of patients [23]. There are few studies focusing on allograft reconstruction following resection of bone tumours using 3D-Printed cutting guides reporting that the achievement of bone union in osteotomy sites was 75–92.3%, with a median time to bone union of 4.8 months [11, 22, 24]. A monocentric retrospective study analysing 6 patients with mean age at surgery of 30.8 years (range, 18–60 years) with malignant primary bone tumour of the diaphysis of femur or tibia who underwent reconstruction with MBA shaped by GSIs following resection using a 3D-printed PSI, showed that bone union was achieved in nine of 12 (75%) of osteotomy sites with a median time of 4.8 months (range 2.3–8.3 months) [22]. In our cohort, bone union rate was achieved in 92.9% of osteotomy sites, with metaphyseal junctions healing earlier than diaphyseal junctions. The high union rate observed may be partially explained by improved host-graft congruency achieved through precise geometric transfer of planned resection planes to the actual ones.

### Complications

Previous retrospective studies reported complications in patients who underwent intercalary resection of malignant bone tumours of the femur and tibia with reconstruction using intercalary MBA, with complication risk ranging from 25.5% to 45.7%, including fracture (11.8%−28.6%), non-union (8.6%−23.5%), and deep infection (5.8%−17.6%) [25, 26]. No previous studies analysed the complication of intercalary resection and reconstruction with MBA in children with malignant bone tumours of lower limb treated using in-house 3D-printed patient-specific cutting guide, with only one monocentric retrospective study analysing six adult patients with malignant primary bone tumour of the diaphysis of femur or tibia, reporting five complications in six patients including three non-union (50%) and two implant failure (33.3%) [11]. In our series, only one complication (non-union in one out of 14 osteotomy sites) was observed and successfully treated adding bone autografts from iliac bone, with no other complications. Although the small sample size precludes definitive conclusions, these preliminary findings suggest that accurate osteotomies and improved host–graft contact may help reduce risk of inadequate margins or non-union. Moreover, the absence of procedure-related issues implies that in-house cutting guides do not introduce additional risks and that their use is not associated with an increased incidence of infection.

### Functional outcomes

Previous studies focusing on joint‑sparing tumour resection of malignant bone tumours of the femur and tibia and reconstruction with MBA reported mean MSTS score of 26–27 [25, 26]. A monocentric retrospective study analysing 35 patients who underwent joint-preserving resection of osteosarcoma and MBA reconstruction of the distal femur and proximal tibia showed mean functional score of 26 [25]. Our favourable functional outcome is consistent with previous studies, with the median MSTS score being 30, showing that intercalary reconstruction may be associated with very good functional results in children due to the preservation of adjacent joints.

In conclusion, in-house 3D-printed patient-specific cutting guides for tumour resection and intercalary graft reconstruction with MBA demonstrated high accuracy in achieving negative margins and precise host-graft matching in children undergoing intercalary reconstruction for malignant bone tumours of the lower limb. In this paediatric oncologic setting, an integrated in-house 3D-printing workflow represents a structured and reproducible approach to enhance surgical precision reducing surgical time and possible complications such as non-union.

## Data Availability

No datasets were generated or analysed during the current study.
